# Supplementary material for: Evaluation of a Cardiovascular Systems Model for Design and Analysis of Hemodynamic Safety Studies
Source: Pharmaceutics. 2023 Apr 7;15(4):1175. doi: 10.3390/pharmaceutics15041175 (PMC10143046; doi:10.3390/pharmaceutics15041175)
Supplement: Supplementary file 1 [file pharmaceutics-15-01175-s001.zip › pharmaceutics-2186186-supplementary.pdf]

pharmaceutics

Supplementary Materials: Evaluation of a Cardiovascular Systems Model for Design and Analysis of Hemodynamic safety Studies

Yu Fu, Nelleke Snelder, Tingjie Guo, Piet H. van der Graaf and J. G. Coen van Hasselt

Supplemental material 1

```
$PROBLEM PK-PD

$INPUT ID TIME DV AMT DV_FLAG

$DATA dat.csv IGNORE=@

$SUBROUTINES ADVAN13    TOL = 6

$MODEL
COMP(PK, DEFDOSE)
COMP(HR)
COMP(SVT)
COMP(TPR)

$PK
;; PK
K = THETA(1)

;; Emax model
EC50 = THETA(2)
EMAX = THETA(3)

;; Feedback
FB = THETA(4)

;; Baseline
BSLHR    = THETA(5)*EXP(ETA(1))
BSLMAP = THETA(6)*EXP(ETA(2))
BSLCO    = THETA(7)*EXP(ETA(3))

BSLSV    = BSLCO/BSLHR
BSLTPR = BSLMAP/BSLCO

;; Kout
KOUTHR    = THETA(8)
KOUTSV    = THETA(9)
KOUTTPR = THETA(10)

;; HR on SV
HRSV = THETA(11)

;; Kin
KINHR    = KOUTHR*BSLHR/(1 - FB*BSLMAP)
KINSV    = KOUTSV*BSLSV/(1 - FB*BSLMAP)
```

```
KINTPR = KOUTTPR*BSLTPR/(1 - FB*BSLMAP)

;; Initial values
A_0(1) = 0
A_0(2) = BSLHR
A_0(3) = BSLSV
A_0(4) = BSLTPR

PER = 24
HOR1 = THETA(12)
AMP1 = THETA(13)
HOR2 = THETA(14)
AMP2 = AMP1*THETA(15)
PI = 3.1415926535

$DES

SV = A(3)*(1 - HRSV*LOG(A(2)/BSLHR))
CO = A(2)*SV
MAP = CO*A(4)

CSHR = AMP1*COS(1*2*PI*(T+HOR1)/PER)
CSTPR= AMP2*COS(1*2*PI*(T+HOR2)/PER)

;; PK
DADT(1) = -K*A(1)

;; HR
DADT(2) = KINHR*(1+CSHR)*(1 - FB*MAP)*(1 + EMAX*A(1)/(EC50+A(1))) - KOUTHR*A(2)

;; SVT
DADT(3) = KINSV*(1 - FB*MAP) - KOUTSV*A(3)

;; TPR
DADT(4) = KINTPR*(1+CSTPR)*(1 - FB*MAP) - KOUTTPR*A(4)


$ERROR

COIPRED = A(2)*A(3)*(1 - HRSV*LOG(A(2)/BSLHR))
MAPIPRED = COIPRED*A(4)

IF (F.EQ.0)    IPRED = 1

IF (DV_FLAG.EQ.2) IPRED = A(2)          ;;   HR
IF (DV_FLAG.EQ.3) IPRED = COIPRED       ;;   CO
IF (DV_FLAG.EQ.4) IPRED = MAPIPRED      ;;   MAP

W = IPRED

IF (DV_FLAG.EQ.2)      Y = IPRED *(1+EPS(1))
IF (DV_FLAG.EQ.3)      Y = IPRED *(1+EPS(2))
IF (DV_FLAG.EQ.4)      Y = IPRED *(1+EPS(3))
```

```
IRES = DV-IPRED
IWRES = IRES/W

$THETA
(0.17325)FIX; TH1 K

(0, 100)      ; TH2 EC50
(-1,1)           ; TH3 EMAX

(0, 0.0029) FIX; TH4 FB

(0, 310)      FIX; TH5 BSLHR
(0, 155)      FIX; TH6 BSLMAP
(0, 69)       FIX; TH7 BSLCO

(0, 11.6)     FIX; TH8 KOUTHR
(0, 0.126)    FIX; TH9 KOUTSV
(0, 3.58)     FIX; TH10 KOUTTPR

(0, 0.312)    FIX; TH11 HRSV

(0, 8.73)     FIX; TH12 HOR HR
(0, 0.0918) FIX; TH13 AMP HR
(0, 19.3)     FIX; TH14 HOR TPR
(1   FIX)       ; TH15 AMP TPR ratio

$OMEGA
(0.00372)     ; IIV_BSLHR
(0.00137)     ; IIV_BSLMAP
(0.0515)      ; IIV_BSLCO

$SIGMA
0.006084      ; Prop.err on HR
0.004761      ; Prop.err on CO
0.0036         ; Prop.err on MAP


$EST METHOD=1 INTER MAXEVAL=5000 NOABORT SIG=3 PRINT=5 POSTHOC
$COV

$TABLE ID TIME DV AMT DV_FLAG MDV EVID IPRED PRED ONEHEADER NOPRINT FILE=sdtab001
```

Table S1. Simulation scenarios of the practical identifiability analysis [a].

	Number of animals	MoA of the Original model	EC_{50} (ng/ml)	E_{max}	Number of doses[b]	Observations	Observation duration	MoA of the Alternative model
Quantification of system- and drug-specific parameters	5	HR	100	-1	3 / 5	HR, CO, MAP / HR, MAP	24h	HR
	5	SV	100	-1	3 / 5	HR, CO, MAP / HR, MAP	24h	SV
	5	TPR	100	-1	3 / 5	HR, CO, MAP / HR, MAP	24h	TPR

Identification of MoA with different drug effects	5	HR	100 / 1000	-1 / 1 / 10	3	HR, CO, MAP / HR, MAP	24h	None / HR / SV / TPR
	5	SV	100 / 1000	-1 / 1 / 10	3	HR, CO, MAP / HR, MAP	24h	None / HR / SV / TPR
	5	TPR	100 / 1000	-1 / 1 / 10	3	HR, CO, MAP / HR, MAP	24h	None / HR / SV / TPR
Identification of MoA with different observation duration	5	HR	100 / 100000	-1	3	HR, CO, MAP / HR, MAP	3 / 6 /12/ 24h	None / HR / SV / TPR
	5	SV	100 / 100000	-1	3	HR, CO, MAP / HR, MAP	3 / 6 /12/ 24h	None / HR / SV / TPR
	5	TPR	100 / 100000	-1	3	HR, CO, MAP / HR, MAP	3 / 6 /12/ 24h	None / HR / SV / TPR
Identification of MoA with different number of animals	3 / 4 / 5	HR	100 / 100000	-1	3	HR, CO, MAP / HR, MAP	24h	None / HR / SV / TPR
	3 / 4 / 5	SV	100 / 100000	-1	3	HR, CO, MAP / HR, MAP	24h	None / HR / SV / TPR
	3 / 4 / 5	TPR	100 / 100000	-1	3	HR, CO, MAP / HR, MAP	24h	None / HR / SV / TPR

[a] HR: heart rate; SV: stroke volume; CO: cardiac output; TPR: total peripheral resistance; MAP: mean arterial pressure; MoA: mode of action.

[b] three ascending doses: 0.1 mg/kg, 1 mg/kg and 10 mg/kg *i.v.* bolus on day 1, 2 and 3; five ascending doses: 0.1 mg/kg, 0.3 mg/kg, 1 mg/kg, 3 mg/kg and 10 mg/kg *i.v.* bolus on day 1 to 5.

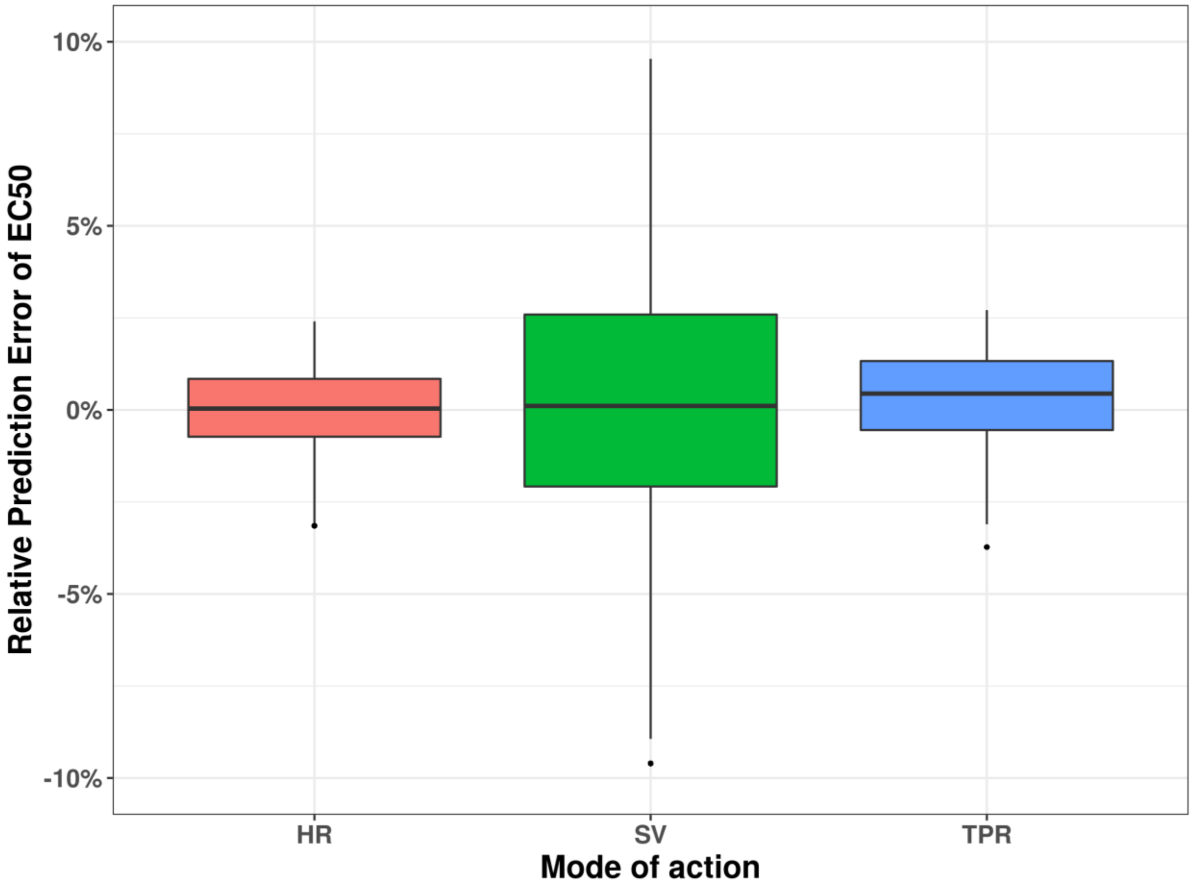

Figure S1. Comparison of final estimates of EC_{50} in models with unfixed and fixed system-specific parameters.

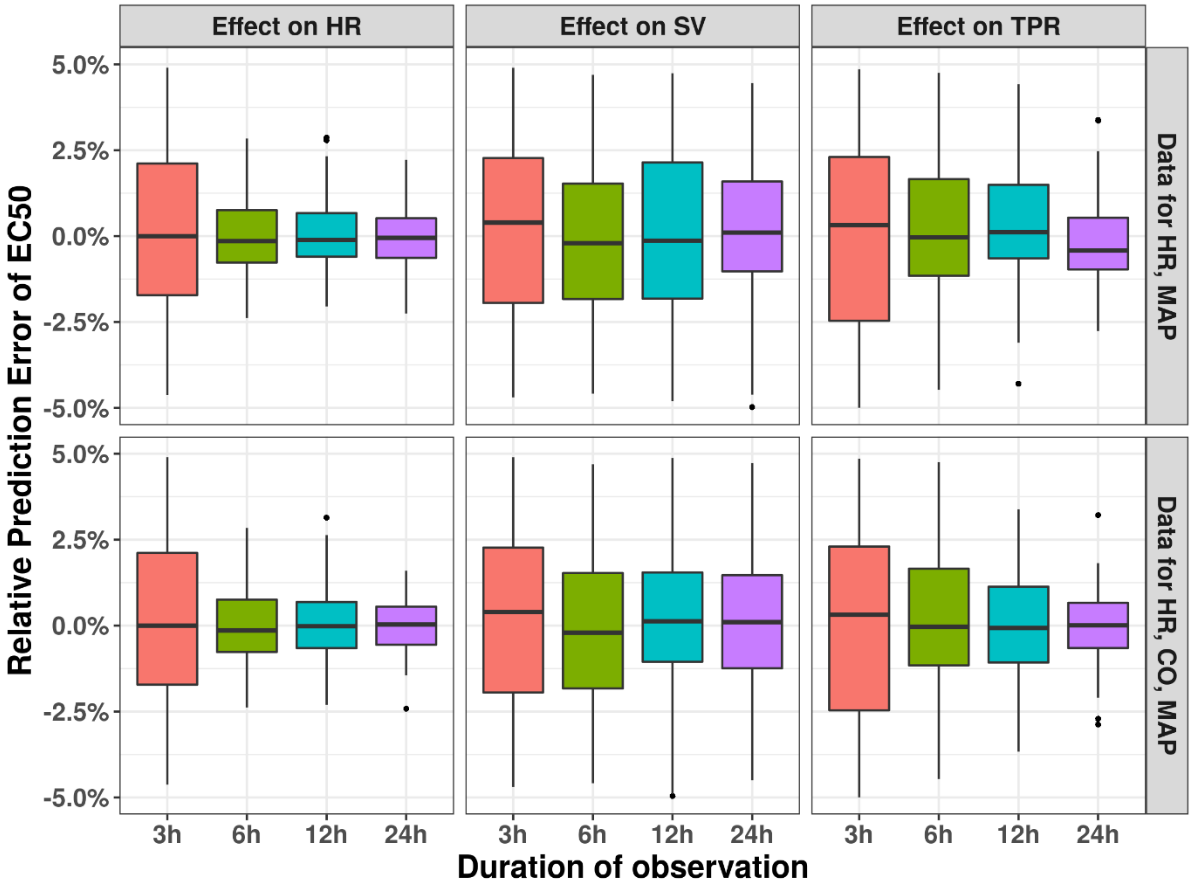

Figure S2. Relative prediction error of EC_{50} in SSE analyses with observations of HR and MAP or observations of HR, CO and MAP within 3h, 6h, 12h and 24h, while Emax was fixed as -1 and EC_{50} was fixed as 100 ng/ml in the original model.The relative prediction error is defined as the ratio of the difference of the parameters' estimates and the parameters' true values over the parameters' true values.

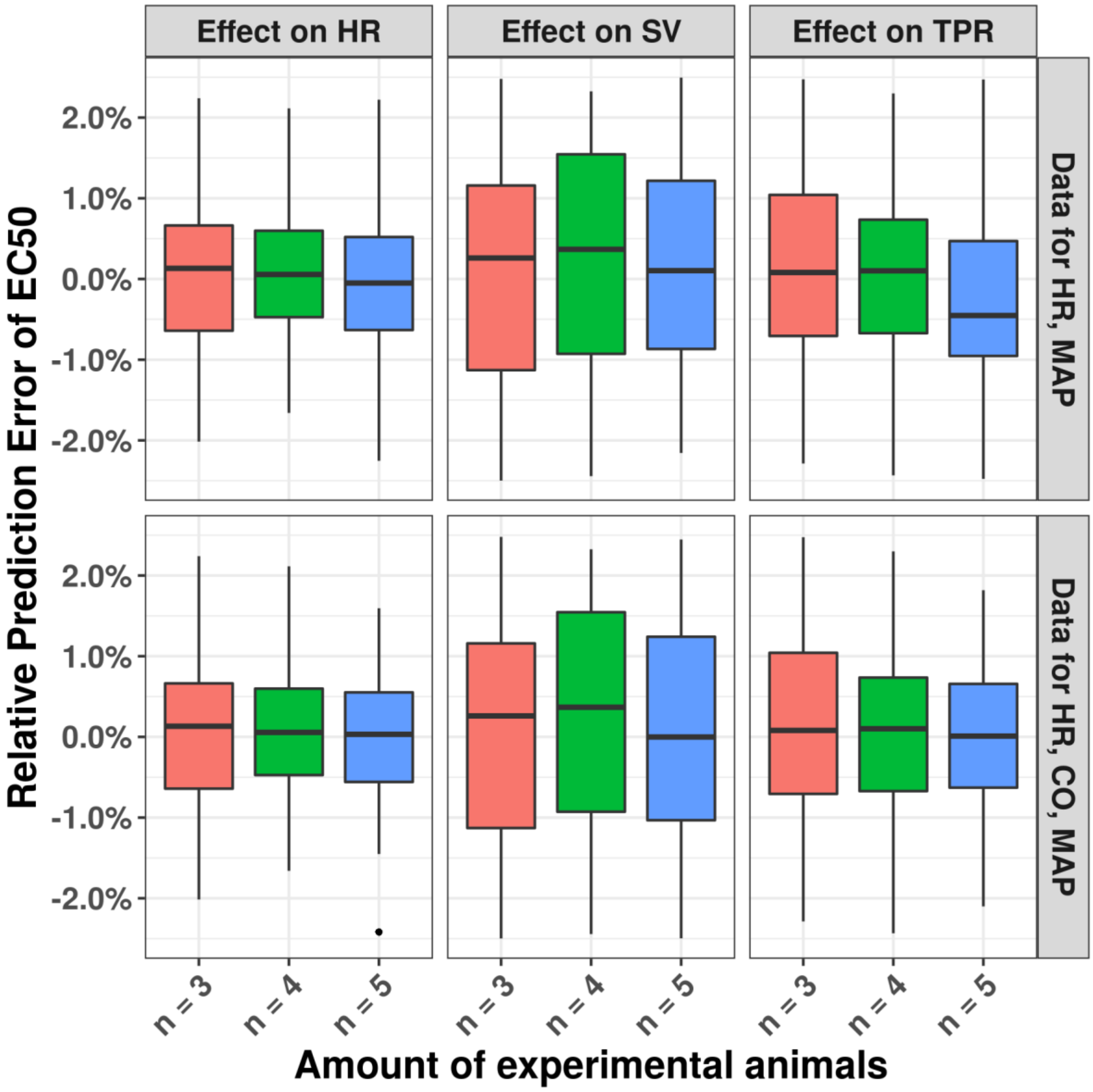

Figure S3. Relative prediction error of EC_{50} in SSE analyses using data for 3, 4, or 5 animals with observations of HR and MAP or observations of HR, CO and MAP, while Emax was fixed as -1 and EC_{50} was fixed as 100 ng/ml in the original model. The relative prediction error is defined as the ratio of the difference of the parameters' estimates and the parameters' true values over the parameters' true values.
